# A Two-Step Screening, Measurement of HbA1c in Association with FPG, May Be Useful in Predicting Diabetes

**DOI:** 10.1371/journal.pone.0036309

**Published:** 2012-04-27

**Authors:** Kyoko Nomura, Kazuo Inoue, Kimihiko Akimoto

**Affiliations:** 1 Teikyo University School of Medicine, Department of Hygiene and Public Health, Tokyo, Japan; 2 Teikyo University School of Medicine, Department of Community Medicine, Tokyo, Japan; 3 Yuport, Medical Checkup Center, Tokyo, Japan; Virgen Macarena University Hospital, School of Medicine, Spain

## Abstract

**Backgrounds:**

We compared the usefulness of fasting plasma glucose (FPG), or hemoglobin A1c (HbA1c), or both in predicting type 2 diabetes.

**Methods:**

This retrospective cohort study investigated 9,322 Japanese adults (4,786 men and 4,536 women), aged 19–69 yrs, free of diabetes at baseline. Usefulness was assessed by predictive values (PV), sensitivity, specificity, and the area under the receiver operating characteristic curve (AUROC) maximised under the best cut-off point.

**Results:**

During the average 6 years of follow-up, 221 men (4.6%) and 92 women (2%) developed diabetes. The best cut-off points for FPG (i.e., 5.67 mmol/l for men and 5.5 mmol/l for women) gave excellent AUROC, and the highest positive PV (13% for men and 9% for women) in predicting diabetes. In high risk subjects with FPG 6.1–6.9 mmol/l, 119 men (26.8%) and 39 women (28.3%) developed diabetes. Under the best cut-off points of FPG 6.39 mmol/l and A1c 5.8, AUROC and positive PV for FPG slightly decreased indicating FPG became less useful and were statistically indistinguishable from those for HbA1c in men. In fact, HbA1c was the most useful in women: HbA1c of 6.0% gave the highest positive likelihood ratio of 2.74 and larger AUROC than did FPG. Although AUROC for HbA1c was acceptable and indistinguishable from that for the combined use, HbA1c had higher specificity and positive LR than did the combined use.

**Conclusions:**

This study demonstrated that FPG was the most useful to predict diabetes in the general population. However, in subjects with FPG 6.1–6.9 mmol/l, FPG became less useful and diagnostic performance of FPG was indistinguishable from that of HbA1c in men whereas HbA1c was the most useful in women. Thus, a two-step screening, measurement of HbA1c in association with FPG, may be useful in predicting diabetes.

## Introduction

The prevalence of type 2 diabetes is increasing at an alarming rate. Current projections suggest that the absolute number of cases worldwide may double over the next two decades [1]. Diabetes causes long-term complications affecting the eyes, kidneys, and the nervous system and leads to the development of micro- and macro-vascular diseases. The speed of this progression is rapid; people with newly diagnosed diabetes may already have retinopathy. Therefore, early detection and intervention in diabetes is now considered one of the most important public health agendas.

Fasting plasma glucose (FPG) is a simple, easy, inexpensive, and widely available to general population and has been most frequently used to identify subjects at high risk of diabetes. Hemoglobin A1c (HbA1c), an indirect measure of mean blood glucose over the previous 2–3 months, does not require fasting, and is more reproducible than FPG [2]. The 2-h plasma glucose after oral glucose tolerance test (OGTT) is also useful to identify subjects at impaired glucose tolerance. However, the OGTT is not common in clinical practice, because it may be difficult to perform and where the cost and demands on participants' time may be excessive [3].

Due to the recent advancement of HbA1c measurement, the American Diabetes Association (ADA) report in 2009 [2] advocated that, the diagnosis of diabetes may be conveniently based on A1c≥6.5%. However, the results of previous studies have been inconsistent between A1c and FPG as to which test yields better screening/predicting performance [4]–[11]. The inconsistency is mainly due to the following two reasons. First, these previous studies used methodologically two different models, either a prognostic model or a diagnostic model or both. The former usually estimates the risk of developing a disease outcome (i.e., the odds ratio, or alternatively, the rate ratios or hazard ratio) whereas the latter discriminates subjects with the disease state from those without and typically uses receiver operating characteristic (ROC) curves. Because the number of an odds ratio has little impact on the ROC curve analyses [12], the mixed use of these two different models might have caused the inconsistent results. Second, among the studies that used diagnostic models, the evaluation was typically based on the efficacy measures such as sensitivity, specificity, and ROC and paid less attention to positive predictive values. Positive PV which increases as prior probability of disease (i.e., prevalence) increases is a powerful indicator of usefulness of a test [13]. In this study, using a diagnostic model, we investigated positive PV as well as the efficacy measures and compared usefulness of three screening tests of FPG, A1c, and the combined use in general population and high risk individuals with FPG 6.1–6.9 mmol/l (110–125 mg/dl).

## Methods

The data was obtained from those who received the complete medical check-up at the Japan Post affiliated health centre. The complete medical check-up offers comprehensive cancer screening which is not covered by the annual health check-ups enforced by law. Those who underwent the medical check-ups were nearby residents, workers related to the Japan Post, and policy holders of postal life insurance provided by the Japan Post.

In this study we set the 4-year baseline period to be between April 1998 and March 2002 and the 4-year follow-up period between April 2002 and March 2006. Study subjects were those who took the check-up at both the baseline period and follow-up period, yielding 11,129 persons. Further, among subjects at baseline, we excluded those who already developed diabetes including 129 subjects with known diabetes, 410 subjects with FPG≥7 mmol/l, and 140 subjects with HbA1c≥6.5%. In addition, 715 elderly subjects aged 70 years and above were excluded because a large-scale epidemiological study reported that older age was significantly associated with having a higher HbA1c level even among healthy individuals [14]. Finally, after we excluded those whose follow-up period was 2 years or shorter, 9,322 Japanese adults (4,786 males, 51%) aged 19–69 years became our study subjects for analyses.

In accordance with the Private Information Protection Law, information that might identify subjects was safeguarded by the Medical Checkup Center. This study was approved by the review board of Yuport Medical Checkup Center and a written informed consent for anonymous participation in epidemiological research was obtained at every evaluation.

All procedures were performed using the same protocols during the baseline and follow-up periods, including blood tests. Height and weight were measured to calculate body mass index (BMI). Blood pressure was measured by trained nurses using a sphygmomanometer. Blood samples were obtained after overnight fasting and analyzed at the Center's laboratory. Triglycerides and total and high-density lipoprotein (HDL) cholesterol were measured using enzymatic methods (reagents supplied by Daiichi Pure Chemicals, Tokyo, Japan). FPG and HbA1c were measured using a Toshiba TBA-40FR auto analyzer (Toshiba Medical Systems, Tokyo, Japan). Plasma glucose was measured using the hexokinase-G6PD method (Denka Seiken, Niigata, Japan). HbA1c was measured using the latex immuno-agglutinin method (Determiner hemoglobin HbA1c; Kyowa Medex, Tokyo, Japan). Comparison of the Japan Diabetes Society primary standard material using an assay by the Anchor Laboratory of the National Glycohemoglobin Standardization Program (NGSP) in the USA revealed that the NGSP value (%) = JDS value (%)+0.4% [15]. Thus, our results were reported using converted NGSP values. NGSP alignment is equivalent to the Diabetes Control and Complications Trial alignment.

Diabetes was defined according to the 2010 American Diabetes Association (ADA) criteria [16]: FPG≥7.0 mmol/l, HbA1c values ≥6.5%, or both, or treatment by oral antidiabetic drugs or insulin. We defined high risk subjects as those with 6.1–6.9 mmol/l (110–125 mg/dl).

Usefulness of a test was assessed by sensitivity, specificity, likelihood ratios (LR), AUROC, and PV. The best cutoff point known to be closest to the upper left-hand corner of the ROC curve was determined where the test characteristics were maximized. In a trade-off between sensitivity and specificity, sensitivity was prioritized over specificity as much as possible for the purpose of screening. Positive/negative PV is defined as the proportion of those with a positive/negative test result who actually has/does not have disease. When the pretest probability of disease is high, positive PV increases [13]. This means, the increase of positive PV indicates that a larger number of people at risk will be detected and thus is used as a measure of usefulness.

Basic characteristics of study subjects are presented as mean and standard deviation (SD) or median with inter-quartile range (IQR) according to the distribution of each variable. Age-adjusted ROC curves were drawn by logistic regression models, with FPG and HbA1c treated as continuous variables. Regression lines were separately fitted between the newly identified diabetics and HbA1c, FPG, and the combination of FPG and HbA1c [8]. The age-adjusted AUROC and the 95% CI were calculated by the Delong method. According to Hosmer and Lemeshow [17], an AUROC value between 0.7 and 0.8 is considered “acceptable,” and one between 0.8 and 0.9 “excellent” discrimination.

Test characteristics were calculated using SAS software (version 9.12, Cary, NC, USA), and the AUROC was calculated using STATA software (version 11, College Station, TX, USA).

## Results

During the follow-up period (average of 6 years), 221 men (4.6%) and 92 women (2%) in the entire subject population developed type 2 diabetes. Among subjects whose FPG level was between 6.1–6.9 mmol/l during the baseline period, 119 men (26.8%) and 39 women (28.3%) developed type 2 diabetes. Table 1 shows baseline characteristics in the entire subject population and subjects with FPG 6.1–6.9 mmol/l according to gender. In the entire subject population, mean FPG was statistically higher in men than in women and mean HbA1c was higher in women than in men. In subjects with FPG of 6.1–6.9 mmol/l, mean HbA1c was higher in women than in men but FPG was not statistically different between gender.

**Table 1:** Baseline Characteristics^a^.

	All			Subjects with FPG 6.1–6.9 mmol/l		
	Men (n = 4786)	Women (n = 4536)	p	Men (n = 444)	Women (n = 138)	p
Age(yrs)	50±11	53±10	<.0001	54±8	57±7	<.0001
Body Mass Index (kg/m2)	23.6±2.8	22.2±3.0	<.0001	24.8±2.9	24.1±3.9	0.887
Fasting plasma glucose (mmol/l)	5.41±0.49	5.10±0.46	<.0001	6.39±0.24	6.37±0.24	0.450
A1c(%)	5.36±0.39	5.38±0.40	0.008	5.68±0.39	5.80±0.40	0.002
HDL cholesterol (mmol/l)	1.38±0.35	1.68±0.38	<.0001	1.33±0.31	1.54±0.37	<.0001
Triglycerides† (mmol/l)	1.28 (0.92, 1.84)	0.91 (0.68, 1.27)	<.0001	1.51 (1.09, 2.07)	1.20 (0.89, 1.70)	<.0001
Total cholesterol (mmol/l)	5.12±0.87	5.37±0.92	<.0001	5.33±0.93	5.60±1.00	0.006
Uric acid(mmol/l)	0.10±0.02	0.08±0.02	<.0001	0.11±0.02	0.09±0.02	<.0001
Systolic blood pressure (mmHg)	126±17	120±18	<.0001	133±18	132±15	0.880
Diastolic blood pressure (mmHg)	77±11	72±11	<.0001	81±11	79±10	0.209

a Presented as mean ±SD.

† Triglycerides is presented with median (25%, 75%) because of the skewed distribution.

**Figure 1:**
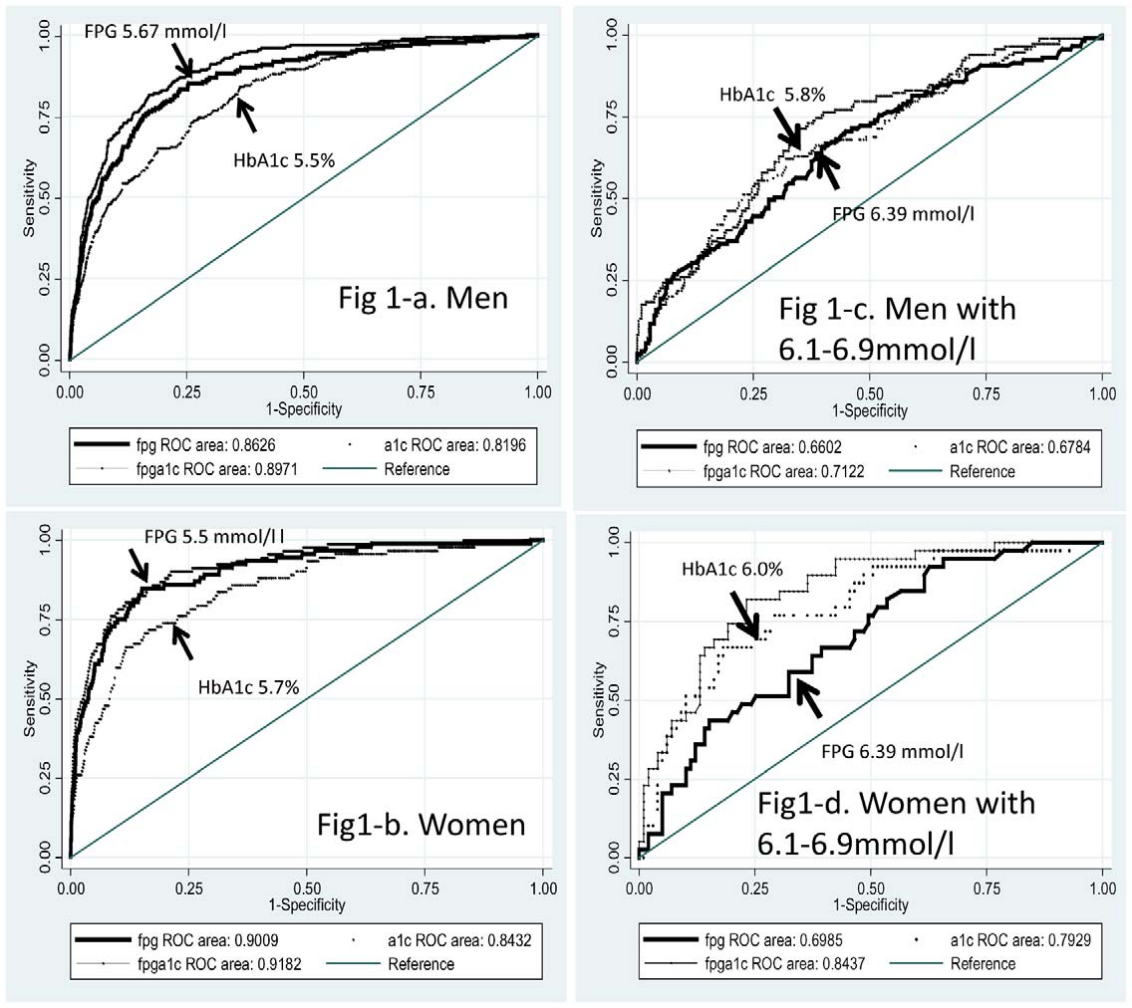
The best cut-off points shown in the receiver operating characteristic curve, conducted in Japan, 1998–2006. Abbreviations:A1c, HbA1c; FPG, fasting plasma glucose, fpgplusA1c, the combined use of FPG and HbA1c. Figure 1A. Men (n = 4786). Figure 1B. Women (n = 4536). Figure 1C. Men with 6.1–6.9 mmol/l (n = 444). Figure 1D. Women with 6.1–6.9 mmol/l (n = 138).

The best cut-off points, closest to the left upper corner of AUROC were 5.67 mmol/l for FPG and 5.5% for HbA1c in men (Figure 1A), and 5.5 mmol/l for FPG and 5.7% for HbA1c in women (Figure 1B). In subjects with FPG 6.1–6.9 mmol/l, the cut-off points were 6.39 mmol/l for FPG and 5.8% for HbA1c in men (Figure 1C) and 6.39 mmol/l for FPG and 6.0% for HbA1c in women (Figure 1D). The AUROC for FPG (0.86, 95%CI:0.84–0.89 for men and 0.90, 95%CI:0.87–0.94 for women, Table 2) was statistically greater than that for HbA1c in both men and women (0.82, 95%CI:0.79–0.85 for men and 0.84, 95%CI:0.80–0.89 for women, Table 2). However in subjects with FPG of 6.1–6.9 mmol/l, the AUROC for HbA1c (0.79, 95%CI: 0.71–0.88, Table 3) was statistically greater than that for FPG (0.70, 95%CI: 0.61–0.79, Table 3) in women.

**Table 2:** Test Characteristics (95% Confidence Interval) maximized under the Best Cut-off Point in the entire subject population.

	Sen	Spec	PPV	NPV	LR+	LR−	Age-adjusted AUROC
Men (n = 4786, diabetes: 4.6%, cut off points: FPG 5.67 mmol/l, A1c 5.5)							
FPG	0.85 (0.80–0.89)	0.73 (0.72–0.75)	0.13 (0.12–0.15)	0.99 (0.98–0.99)	3.20 (2.97–3.44)	0.20 (0.15–0.28)	0.86 (0.84–0.89)
HbA1c	0.84 (0.79–0.89)	0.63 (0.62–0.65)	0.10 (0.09–0.11)	0.98 (0.98–0.99)	2.29 (2.14–2.46)	0.25 (0.18–0.34)	0.82 (0.79–0.85)
FPG plus HbA1c	0.97 (0.94–0.99)	0.51 (0.49–0.52)	0.09 (0.08–0.10)	0.99 (0.99–1.00)	1.98 (1.91–2.06)	0.05 (0.02–0.12)	0.90 (0.88–0.92)
Women (n = 4536, diabetes: 2.0%, cut off points: FPG 5.5 mmol/l, A1c 5.7)							
FPG	0.85 (0.75–0.91)	0.82 (0.81–0.84)	0.09 (0.07–0.11)	0.99 (0.99–1.00)	4.81 (4.33–5.37)	0.19 (0.11–0.30)	0.90 (0.87–0.94)
HbA1c	0.76 (0.66–0.84)	0.76 (0.66–0.84)	0.06 (0.05–0.08)	0.99 (0.99–0.99)	3.16 (2.79–3.59)	0.32 (0.22–0.45)	0.84 (0.80–0.89)
FPG plus HbA1c	0.94 (0.86–0.97)	0.66 (0.64–0.67)	0.05 (0.04–0.07)	0.99 (0.99–0.99)	2.73 (2.55–2.92)	0.10 (0.05–0.22)	0.92 (0.89–0.95)

Abbreviations: CI, Confidence Interval; FPG, Fasting Plasma Glucose; LR+, Positive Likelihood Ratio; LR−, Negative Likelihood Ratio; NPV, Negative Predictive Value; PPV, Positive Predictive Value; Sen, Sensitivity; Spec, Specificity.

**Table 3:** Test Characteristics (95% Confidence Interval) maximized under the Best Cut-off Point in Subjects with FPG 6.1–6.9 mmol/l.

	Sen	Spec	PPV	NPV	LR+	LR−	Age-adjusted AUROC
Men (n = 444, diabetes: 26.8%, cut off points: FPG 6.39 mmol/l, A1c 5.8)							
FPG	0.65 (0.56–0.74)	0.60 (0.55–0.66)	0.38 (0.31–0.45)	0.83 (0.77–0.87)	1.65 (1.37–1.99)	0.57 (0.44–0.74)	0.67 (0.60–0.72)
HbA1c	0.63 (0.54–0.72)	0.64 (0.58–0.69)	0.39 (0.32–0.46)	0.83 (0.77–0.87)	1.74 (1.42–2.12)	0.58 (0.46–0.74)	0.68 (0.62–0.73)
FPG plus HbA1c	0.83 (0.75–0.89)	0.42 (0.37–0.48)	0.35 (0.29–0.40)	0.87 (0.81–0.92)	1.44 (1.27–1.63)	0.40 (0.27–0.60)	0.71 (0.66–0.77)
Women (n = 138, diabetes: 28.3%, cut off points: FPG 6.39 mmol/l, A1c 6.0)							
FPG	0.62 (0.45–0.76)	0.64 (0.53–0.73)	0.40 (0.28–0.54)	0.81 (0.70–0.89)	1.69 (1.18–2.43)	0.60 (0.40–0.91)	0.70 (0.61–0.79)
HbA1c	0.69 (0.52–0.82)	0.75 (0.65–0.83)	0.52 (0.38–0.66)	0.86 (0.77–0.92)	2.74 (1.84–4.08)	0.41 (0.25–0.66)	0.79 (0.71–0.88)
FPG plus HbA1c	0.92 (0.78–0.98)	0.48 (0.37–0.58)	0.41 (0.31–0.52)	0.94 (0.82–0.98)	1.76 (1.43–2.16)	0.16 (0.05–0.49)	0.84 (0.77–0.91)

Abbreviations: CI, Confidence Interval; FPG, Fasting Plasma Glucose; LR+, Positive Likelihood Ratio; LR−, Negative Likelihood Ratio; NPV, Negative Predictive Value; PPV, Positive Predictive Value; Sen, Sensitivity; Spec, Specificity.

Table 2 shows test characteristics maximized under the best cut-off point in the entire subject population. In men, the combined use of FPG and A1c had the largest AUROC (0.90, 95%CI:0.88–0.92). However, the AUROC for FPG had also excellent discrimination (0.86, 95%CI: 0.84–0.89). In addition, FPG had higher specificity (0.73, 95%CI: 0.72–0.75) and positive PV (0.13, 95%CI: 0.12–0.15) compared to those for the combined use (0.51, 95%CI: 0.49–0.52 and 0.09, 95%CI: 0.08–0.10, respectively). This indicates that FPG alone than the combined use is more useful in men.

In women, both FPG alone and the combined use had the largest AUROC (0.90, 95%CI: 0.87–0.94 vs. 0.92, 95%CI: 0.89–0.95). But among there two, FPG alone had higher specificity (0.82, 95%CI: 0.81–0.84) and positive PV (0.09, 95%CI: 0.07–0.11) compared to those for the combined use (0.66, 95%CI: 0.64–0.67 and 0.05, 95%CI: 0.04–0.07, respectively). This indicates that FPG alone than the combined use is more useful also in women.

Table 3 shows test characteristics maximized under the best cut-off point in high risk subjects with FPG 6.1–6.9 mmol/l. In men, positive PV, and AUROC as well as other efficacy measures except for specificity and negative LR were not statistically different among the three screening tests of FPG, HbA1c and the combined use. The combined use had the least negative LR of 0.4 indicating that the negative result of both FPG and HbA1c did not exclude diabetes with sufficient certainty. In addition, the combined use had the least specificity. Thus, FPG or HbA1c alone was thought to be more useful than the combined use in men in this group.

In women, both HbA1c alone and the combined use had the largest AUROC (0.79, 95%CI: 0.71–0.88 and 0.84, 95%CI: 0.77–0.91, respectively). But HbA1c had higher specificity (0.75, 95%CI: 0.65–0.83) and positive LR (2.74, 95%CI: 1.84–4.08), than those for the combined use (0.48, 95%CI: 0.37–0.58 and 1.76, 95%CI: 1.43–2.16, respectively). This indicates that HbA1c alone is the most useful in women.

## Discussion

To summarize our results, FPG was the most useful screening test in predicting diabetes in the entire study population, but in high risk subjects with FPG of 6.1–6.9 mmol/l, FPG became less useful and diagnostic performance of FPG was indistinguishable from that of HbA1c in men whereas HbA1c was the most useful in women.

Previously, several studies based on AUROC analyses have reported that the combined use of HbA1c and FPG had the highest efficacy for diabetes [7]–[9]. Indeed, this study agreed that the combined use had the largest AUROC across the strata but demonstrated that AUROC for FPG also had excellent discrimination in whole men and women. In addition, FPG had the highest positive PV among three comparisons. This indicates that FPG is the most useful test in whole population because it can detect a larger number of individuals with diabetes. Thus, our study suggests that the test with the largest AUROC is not equal to the most useful test.

Our results that measurement of HbA1c in association with FPG is useful in predicting diabetes are consistent with the results of recent studies [18]–[20]. A study conducted by Inoue et al. [18] diagnosed diabetes in 10,042 subjects using FPG and HbA1c, and reported that diabetes diagnosis with FPG levels between 5.6 and 6.9 mmol/L and an elevated HbA1c between 5.5 and 6.4% led to substantial improvements in the risk of progression to diabetes. Another study conducted by Heianza, et al. [19] investigated 6241 subjects and reported that predictive value of progression to diabetes assessed by HbA1c 5.7–6.4% substantially increased in those with impaired fasting glucose (IFG). These studies indicate that in high-risk individuals, diagnostic criteria based on FPG criteria are relatively insensitive, but HbA1c measurement improves the sensitivity of screening. Furthermore, a meta-analysis [20] reported that dysglycaemic individuals were at a roughly five-to-ten times increased risk of diabetes compared with individuals without IFG or impaired glucose tolerance.

Our study had limitations that should be discussed. First, there were 21,885 subjects in total who participated in the complete medical check-ups. Among these, we only included those who underwent the check-ups at both baseline and follow-up periods, which might have caused selection bias. Nevertheless, when comparing baseline characteristics between those who did and did not participate in the follow-up, the mean of age (52.9 vs. 51.8 y/o), BMI (22.9 vs. 22.8 kg/m2), FPG levels (5.27 vs. 5.28 mmol/l), and HbA1c (4.97 vs. 4.95%) were actually comparable between the two groups. Second, FPG and HbA1c in this study were assessed only at baseline and follow-up. The inter- and intra-coefficient variations in glucose values may have caused some random misclassification in glucose categories and thereby influenced our results. Nevertheless, glucose levels in healthy individuals do not fluctuate as observed in diabetic subjects [21]. Furthermore, because our subjects had blood tests right before they underwent gastrofiberscopy and abdominal ultrasound, measurement of FPG in the fasting state was highly reliable. Third, the present study did not use OGTT as a basis for exclusion, which might influence the results. Given that, according to a previous report, FPG alone failed to diagnose 30% of patients with diabetes who were diagnosed by a 2-h plasma glucose test [22], some individuals in our study might have had diabetes at inclusion. Thus, the results of our study require careful attention to interpret the findings. Forth, in high risk men with 6.1–6.9 mmol/l, the optimal screening test is indeterminate between FPG and HbA1c. These two measures had in fact not very high sensitivity (0.65 for FPG and 0.63 for HbA1c) which means that the number of false negative was relatively high. In this regard, we have mandatory health checkup system in Japan where any adults must take periodical glycemic checkup: for every worker by Industrial Safety and Health Act and for the elderly and house wives by Health Promotion Act. Thus, the person who had negative results but diabetes may be more likely to be screened in the subsequent health checkup. Fifth, the result of this study is based on Japanese population and thus may be different in other ethnic groups.

Despite these limitations, the result of this study suggests that FPG may be the most useful in the general population, whereas HbA1c may be more useful in subjects with high risk individuals with 6.1–6.9 mmol/l. Our findings may conflict with the ADA report in 2009 because it advocates that the screening diabetes is based on HbA1c measurement and a repeat HbA1c test should be done for confirmation in asymptomatic patients [3]. Takahashi et al. [23] investigated 16,313 healthy Japanese and reported that the cumulative diabetes incidence at 3 years for those with baseline HbA1c of less than 5.0%, 5.0–5.4%, and 5.5–5.9% was 0.05%, 0.05%, and 1.2%, respectively. The authors further reported that among those with an HbA1c under 6.0%, rescreening at intervals shorter than 3 years identified few individuals (∼1% or less) with an HbA1c ≥6.5%. Thus, this study does not contradict the result of our study suggesting that routine measurement of HbA1c in the general population may not be recommended. Furthermore, Malkani and Mordes [24] suggested that in choosing a diagnostic test for diabetes, the limitations of glucose measurement and HbA1c must be understood; for example, HbA1c assay may not be available in parts of the world and is its greater expense compared to FPG.

In summary, measurement of FPG in the fasting state may be the most useful to predict diabetes in general population., However, our study demonstrated that among high risk subjects with 6.1–6.9 mmol/l, FPG was less useful and the diagnostic performance of FPG was indistinguishable from that of HbA1c in men whereas HbA1c was found to be more useful than FPG in women. Thus, the results of our study suggest a two-step screening in predicting diabetes; firstly the use of FPG is recommended in overall general population and then in high risk subjects with 6.1–6.9 mmol/l, measurement of HbA1c in association with FPG may be useful in predicting diabetes. The results of our study may provide important insight into how to use limited resources for the best health intervention. Given that FPG is less expensive than HbA1c and the local performance of the HbA1c assay is not always available, it is suggested that FPG may be used as a first screening approach, with HbA1c being used for further screening for those at high risk of diabetes. However, it should be noted that screening strategy should provide safety net to screen those with false negative at the initial screening by the subsequent screening.
